# Logical Reasoning, Spatial Processing, and Verbal Working Memory: Longitudinal Predictors of Physics Achievement at Age 12–13 Years

**DOI:** 10.3389/fpsyg.2019.01929

**Published:** 2019-08-21

**Authors:** Ulf Träff, Linda Olsson, Kenny Skagerlund, Mikael Skagenholt, Rickard Östergren

**Affiliations:** Department of Behavioural Sciences and Learning, Linköping University, Linköping, Sweden

**Keywords:** physics, spatial processing, working memory, logical reasoning, reading

## Abstract

To date, few studies have tried to pinpoint the mechanisms supporting children’s skills in science. This study investigated to what extent logical reasoning, spatial processing, and working memory, tapped at age 9–10 years, are predictive of physics skills at age 12–13 years. The study used a sample of 81 children (37 girls). Measures of arithmetic calculation and reading comprehension were also included in the study. The multiple regression model accounted for 24% of the variation in physics achievement. The model showed that spatial processing (4.6%) and verbal working memory (4.5%) accounted for a similar amount of unique variance, while logical reasoning accounted for 5.7% variance. The measures of arithmetic calculation and reading comprehension did not account for any unique variance. Nine percent of the accounted variance was shared variance. The results demonstrate that physics is a multivariate discipline that draws upon numerous cognitive resources. Logical reasoning ability is a key component in order for children to learn about abstract physics facts, concepts, theories, and applying complex scientific methods. Spatial processing is important as it may sub-serve the assembly of diverse sources of visual-spatial information into a spatial-schematic image. The working memory system provides a flexible and efficient mental workspace that can supervise, coordinate, and execute processes involved in physics problem-solving.

## Introduction

In our modern technologically advanced society, it is essential to be scientifically literate, and it has become increasingly important for society that there are individuals willing and able to pursue careers within the science and technology field (Tytler, 2014; Vilia et al., 2017). Despite the importance of acquiring and possessing adequate skills and knowledge in science, few studies have explored the mechanisms underlying children’s basic skills in science. Thus, in an attempt to expand our understanding, the purpose of the present study was to pinpoint the cognitive mechanisms supporting 12-to-13-year-olds’ skills in physics.

Science (i.e., physics, chemistry, and biology) is a complex academic domain that requires the child to not only learn the meaning of scientific concepts but also acquire scientific reasoning skills (Klahr et al., 2011). The former refers to learning about basic scientific facts, theories, and laws whereas the latter refers to learning about and applying scientific methods (i.e., hypothesis generation, experimentation, and evidence evaluation; Klahr et al., 2011; Kuhn, 2011). Thus, several cognitive abilities could hypothetically constitute key components underlying children’s skills in science. The present study focused on three theoretically relevant cognitive abilities that should be related to science: logical reasoning, spatial ability, and verbal working memory.

The selection of the three cognitive abilities is partially founded on research examining the underlying mechanisms of children’s mathematical learning. Evidence shows that working memory, logical reasoning, and spatial ability play unique roles in children’s mathematical achievement and development (Fuchs et al., 2010a,b; Gunderson et al., 2012; Cowan and Powell, 2014; Cirino et al., 2016; Skagerlund and Träff, 2016; Mix et al., 2017). As science and mathematics are two disciplines within the STEM (science, technology, engineering, and mathematics) complex, they may share supporting processes.

### The Role of Logical Reasoning, Spatial Ability, and Verbal Working Memory in Science Achievement

Abundant research shows that logical reasoning is one key component underlying skills in science (e.g., van der Graaf et al., 2015; Vilia et al., 2017; Berkowitz and Stern, 2018). The reason for this dependence upon logical reasoning is rather straightforward as acquiring skills in science involves learning about abstract scientific facts, theories, and applying complex scientific methods (Klahr et al., 2011; Kuhn, 2011). Thus, in order to acquire the complex and abstract content of science, the child must be able to perform logical and abstract thinking (Roth et al., 2015).

Abstract scientific phenomena and concepts (electricity, magnetism, molecular structure, cell structure) are often described and explained by the use of graphs, diagrams, or physical models (Hegarty, 2014; Newcombe, 2016). The interpretation and comprehension of these forms of visual-spatial representations should theoretically place demands on the individual’s spatial processing abilities (Hegarty, 2014; Stieff and Uttal, 2015; Newcombe, 2016; Verdine et al., 2017). Consistent with the assumption, numerous studies on adults show that measures of spatial ability such as mental rotation and spatial visualization are predictive of concurrent and future accomplishment in science (Hegarty and Sims, 1994 spatial visualization; Paper Folding Test; speeded rotation, spatial orientation; Kell et al., 2013 spatial visualization; Kozhevnikov et al., 2007 spatial visualization; Shea et al., 2001; Wai et al., 2009 spatial visualization; Webb et al., 2007 mental rotation; Yoon and Mann, 2017 mental rotation). Furthermore, a few intervention studies provide evidence that training spatial ability can improve science learning in university students (Sorby, 2009; Miller and Halpern, 2013). However, the mechanisms for how spatial ability supports skills and learning in science are still not well understood. Nevertheless, it has been suggested that spatial processing has several different functions during science problem-solving such as creating spatial-schematic images of abstract concepts and performing spatial transformations of these mental images (Kozhevnikov et al., 2002; Miller and Halpern, 2013; Hegarty, 2014). Important spatial transformations entail the ability to mentally rotate images, integrate or relate different components of visual-spatial information, and to decompose images into parts for subsequent individual analysis (Kozhevnikov et al., 2002; Miller and Halpern, 2013; Hegarty, 2014; Verdine et al., 2017).

Similar to mathematics, a number of researchers suggest that working memory constitutes a key mechanism for scientific reasoning (Hegarty and Sims, 1994; Isaak and Just, 1995; Kozhevnikov et al., 2007; Hegarty, 2014). Working memory refers to a multi-purpose mental workspace responsible for coordinating and executing the performance of simultaneous processes such as temporarily storing information, shifting from one strategy or operation to another, and inhibiting activation of irrelevant information (Engle et al., 1992; Shah and Miyake, 1996; Baddeley, 1997). Science is an intricate academic domain involving a complex interplay of reading/language processes, comprehension processes (i.e., logical reasoning), and visual-spatial processes (e.g., transformations; Hegarty, 2014; Bergey et al., 2015; Roth et al., 2015; Newcombe, 2016). As such, it should require a flexible and efficient mental workspace that can supervise, coordinate, and execute the different process involved in science/physics problem-solving (Kozhevnikov et al., 2007). Evidence in support of this assumption is mounting. For example, Gathercole and colleagues have, in a number of studies, observed relationships between science achievement and verbal working memory in children aged 14–15 years (Jarvis and Gathercole, 2003; Gathercole et al., 2004; see also Danili and Reid, 2004) and children aged 11–12 years (St. Clair-Thompson and Gathercole, 2006).

### Multi-Factorial Studies on Children’s Science Skills

To date, few researchers have simultaneously investigated to what extent different cognitive abilities support children’s learning of science. However, a few exceptions to this state of affairs do exist. For example, Rhodes et al. (2014) found in a sample of 56 12- to 13-year-olds that proficiency in biology was supported by visual working memory (16.0%) and planning ability (9.6%), but not inhibition control, or attention shifting. In a later study performed on 63 12- to 13-year-olds, Rhodes et al. (2016) observed that proficiency in chemistry was supported by visual working memory (10.9%) and vocabulary (20.2%), but not inhibition control, attention shifting, or planning ability.

In a recent large-scale study on 5,838 16-year-olds, Donati et al. (2019) investigated the unique contribution of working memory, inhibition control, processing speed, vocabulary, non-verbal logical reasoning, and socio-economic status (SES) to attainment in science. Structural equation modeling showed that working memory (10.3%), non-verbal logical reasoning (0.01%), vocabulary (4.7%), and SES (1.1%) accounted for unique variance in science at age 16 while controlling for previous attainment in science at age 11.

Moreover, Mayer et al. (2014) explored the association between scientific reasoning (understanding the nature of science, understanding theories, designing experiments, and interpreting data) and spatial ability (mental rotation), inhibitory control, problem-solving skills, reading and logical reasoning in 155 10-year-olds (fourth grade). Multiple regression analysis showed that spatial ability accounted for 2.9% unique variance, while problem-solving skills and reading comprehension accounted for 6.7% variance each.

Recently, Hodgkiss et al. (2018) examined to what extent four different categories of spatial abilities (intrinsic-static; intrinsic-dynamic; extrinsic-static; and extrinsic-dynamic; Uttal et al., 2013; Newcombe and Shipley, 2015) and vocabulary contribute to children’s (7–11 years old; *N* = 123) achievements in specific science domains (i.e., physics, biology, chemistry). Multiple regression analyses revealed that all three science domains (biology, chemistry, physics) were supported by vocabulary and spatial abilities, but by somewhat different constellations of spatial abilities. Individual differences in biology scores were accounted for by mental folding (6%), an intrinsic-dynamic skill, and spatial scaling (2%), an extrinsic-static skill. Mental folding also accounted for 4% of the variation in physics scores. Spatial scaling (2%) and embedded figures (3%), an intrinsic-static skill, accounted for variance in chemistry.

In addition to the studies performed on 7- to 16-year-olds, three studies performed on young children (4–6 years old) are relevant for the present study.

For instance, van der Graaf et al. (2016) examined if variation in 100 kindergartners’ (4–5 years old) scientific reasoning (evidence evaluation; experimentation) was supported by verbal working memory, visuospatial working memory, inhibition control, spatial visualization, vocabulary, and grammar. They observed that evidence evaluation was supported by verbal working memory, inhibition control, vocabulary, and grammar but not visuospatial working memory or spatial visualization, while experimentation was only supported by inhibition control.

In a subsequent study on a sample of 100 5- to 6-year-olds, van der Graaf et al. (2018) found that verbal working memory (6.2%) and inhibition (4.8%) provided independent contribution to growth in evidence evaluation but language skills (vocabulary; grammar) did not. Vocabulary (6.8%) and grammar (6.8%), on the other hand, were the only cognitive abilities that accounted for variation in growth in experimentation.

Zhang et al. (2017) tested a sample of 584 6-year-old Chinese children on skills in life sciences (biology), earth and physical sciences (physics; chemistry), language (vocabulary), spatial processing (spatial perception, spatial visualization, and mental rotation), and verbal working memory. Multiple regression analyses revealed that verbal working memory (12.8%), spatial processing (mental rotation; 3.5%), and language (1.3%) contributed to achievement in life sciences, while only language (0.5%) contributed to achievement in earth and physical sciences (physics; chemistry).

In sum, the overall empirical picture regarding the cognitive mechanisms supporting children’s skills in the natural sciences is far from clear. The complicated empirical picture is due to a number of reasons. First, researchers have focused on different domains of science (e.g., biology, chemistry, physics, and generic science) or different aspects of science (e.g., factual knowledge, conceptual reasoning, evidence evaluation, and experimentation). Second, researchers have included somewhat different cognitive abilities in their test batteries, or used different measures to tap the same ability. Third, there is a large variability in age among the samples used in the different studies, ranging from 4–5 years to 16 years. Thus, it is difficult to draw any firm conclusions based on existing research. Hence, more research is required to pinpoint the cognitive mechanisms supporting children’s science learning. However, working memory and language abilities appear to be key components, but their contribution varies among studies from very small (0.5%) to quite large (12.8%). The findings from Mayer et al. (2014), Hodgkiss et al. (2018), and Zhang et al. (2017) corroborate the spatial-science link previously found in adults, by emphasizing the role of spatial abilities in children’s science learning.

### The Current Study

The aim of the present study was to simultaneously investigate to what extent logical reasoning, spatial processing ability, and verbal working memory, tapped in third grade, are independent cognitive abilities underlying children’s future physics skills in sixth grade. Based on prior research and theoretical reasoning, it was hypothesized that all three cognitive abilities should independently account for variation in sixth graders’ understanding of physics.

Unlike prior research that has mainly focused on young adults, this study focused on 12- to 13-year-olds’ physics skills measured by a broad curriculum-based test. In addition to measures of logical reasoning, spatial ability, and verbal working memory, measures of basic arithmetic and reading comprehension were included in the study.

Mathematics and physics are two STEM (science, technology, engineering, and mathematics) disciplines, and as such, they may share certain underlying cognitive processes. Consistent with this, an abundance of research demonstrates a link between mathematics and science proficiency (e.g., Ma and Ma, 2005; Maerten-Rivera et al., 2010; Barnard-Brak et al., 2017). As a matter of fact, when performing science experiments, mathematical tools are used in order to collect (i.e., measure), organize, and analyze data (Batista and Matthews, 2002).

A number of studies also display a link between reading and science proficiency (O’Reilly and McNamara, 2007; Maerten-Rivera et al., 2010; Mayer et al., 2014; Barnard-Brak et al., 2017). This link is theoretically reasonable as almost all classroom teaching is provided verbally or *via* text, reading and language comprehension should play a role in children’s science learning. Moreover, the graphs and diagrams used to describe and explain abstract scientific concepts usually also include textual information that the child has to decode and comprehend (Cromley et al., 2013; Bergey et al., 2015). In view of existing research and theorizing, it was hypothesized that arithmetic ability and reading comprehension should account for unique variation in sixth graders’ understanding of physics.

## Materials and Methods

### Participants

A final sample of 81 Swedish children (37 girls) took part in the study. A letter of consent was distributed in the classroom that the children brought home to the parents. All children with written informed consent from the parents were included in the study. In grade 3, the mean age was 9.62 years (SD = 0.30, min = 9.03, max = 10.33). In grade 6, the mean age was 12.88 years (SD = 0.25, min = 12.34, max = 13.32). All 81 children had Swedish as their native language, no hearing loss, and normal or corrected-to-normal visual acuity. The study did not include children with neuropsychological diagnoses (e.g., ADHD).

This study was approved by the regional ethics committee in Linköping, Sweden (protocol number 33–09).

### General and Test Procedure

In grade 3, 292 children performed the reading test, Raven’s progressive matrices, the arithmetic test, and the mental rotation test in group sessions of 3–5 children. The working memory test was administered during an individual session. All children were tested in familiar rooms at their respective schools. The test instructions were presented orally, and all children received the tests in the same order. During the spring semester in grade 6, 81 children performed the physics test in a classroom session. The test was administered by the classroom teachers as it was part of the national curriculum assessment provided and monitored by the Swedish National Agency for Education. The large attrition is due to that tests of science (biology, chemistry, physics) and social sciences (history, religion, geography, social studies) are optional for the schools to perform, while tests of mathematics, English (first foreign language), and Swedish (native language) are mandatory. A total of 139 (48%) of the 292 children performed tests of either biology (38), chemistry (20), or physics (81).

#### Verbal Working Memory

This test was developed by the first author and has been used in a number of studies on children aged 6–15 years (e.g., Träff et al., 2017a,b,c). The child was presented with sequences of words read by the experimenter, spanning from a minimum of two words to a maximum of seven words. For each word in the sequence, the child was instructed to decide whether the word was an animal or not (by verbally responding either “YES” or “NO”) before advancing to the next word in the sequence. Forty-three percent of the words were animals. Following the presentation of all words within the span, the child was asked to verbally recall the words in the correct serial order. Two such trials were administered for each span, and the child advanced to the next span (e.g., from a span of two to three words) if at least one trial was successfully recalled. Testing concluded when the child failed to correctly recall both trials in a given span. The score, used as an index of verbal working memory, was represented by the longest sequence of correctly recalled words. An additional 0.5 points were awarded if the child correctly recalled the words featured in both trials in her/his longest span size. The possible range of scores was 0–7.5.

#### Spatial Ability

A measure of spatial ability was obtained using a mental rotation task based on alphabetic letters (Rüsseler et al., 2005). Over a total of 16 trials featuring a single letter per trial, a target letter was presented to the left, followed by four adjacent comparison letters to the right. The four comparison letters were rotated in one of six rotation angles (45, 90, 135, 225, 270, and 315°) in the picture-plane, where two comparison letters were visually mirrored (i.e., “incorrect”) instances of the target letter. The child was asked to identify the two non-mirrored (i.e., “correct”) letters matching the target by mentally rotating the comparison stimuli and marking the correct answers with a pen. A maximum score of 16 was awarded if both correct comparison letters were marked in each trial. The dependent measure used was the number of correctly solved trials complete during 120 s. The possible range of scores was 0–16.

#### Non-verbal Logical Reasoning Ability

A shortened version of Raven’s Standard Progressive Matrices (Raven, 1976; design sets B, C, and D; excluding design sets A and E) was used to assess non-verbal logical reasoning ability. Each design set consists of 12 visual pattern designs with one missing piece, and an array of six to eight pieces to be compared with the visual pattern. The child’s task was to select one of the six to eight pieces that appropriately completed the visual design, which was indicated by marking the chosen option on a separate answer sheet. Each child received a test booklet including two practice trials and 36 test items, where a maximum score of 36 was achieved by correctly identifying the missing piece for each trial. After completing the two practice trials, the children completed the 36 trials at their own pace. The possible range of scores was 0–36.

#### Reading Comprehension

This Swedish reading comprehension test was developed by Malmquist (1977) and has been used in a large number of studies on children aged 8–10 years (e.g., Träff and Passolunghi, 2015; Träff et al., 2017b). The child was instructed to read a short story in the form of a fairy-tale. Throughout the text, 20 evenly scattered instances of single words were replaced with a blank space followed by a bracket containing four words. The task was to identify and underline the one out of four words that made the most sense, in terms of story and sentence coherence. The dependent measure was the number of correctly underlined words within a reading time of 4 min. The possible range of scores was 0–20.

#### Arithmetic

This test was developed by the first author and has been used in a number of studies on children aged 8–12 years (e.g., Träff et al., 2017a, in press). The child was instructed to solve six addition and six subtraction problems (e.g., 57 + 42; 545 + 96; 4,203 + 825; 78 − 43; 824–488; 11,305–5,786) in 8 min by means of paper and pencil. The problems were presented horizontally. The children responded in writing. Eight of the 12 problems required carrying or borrowing operations. The dependent measure used was the number of correctly solved problems during 8 min. The possible range of scores was 0–12.

#### Physics in Grade 6

This broad curriculum*-*based test was developed by the Swedish National Agency for Education. It covered many areas of physics, such as electricity (3 problems), gravity (2 problems), optics (2 problems), astrophysics (2 problems), mechanics (1 problem), kinematics (1 problem), density (1 problem), acoustics (1 problem), thermodynamics (1 problem), and magnetism (1 problem). The test consisted of 15 problems; some of them included subproblems resulting in a maximum score of 38 points. The problems featured either fixed response options or open answers. On some problems, the children were required to answer by drawing an illustration of a solution (e.g., a picture of a bulb and battery – “Draw cords so that there is a connection so the lamp is on”). Pictorial information was used in 12 of the 15 problems. The children were allowed 60 min to solve all 15 problems. The possible range of scores was 0–38.

## Results

Descriptive statistics (means, standard deviations, reliabilities, correlations) for the six measures are presented in Table 1. All five predictors were significantly correlated with physics skills.

**Table 1:** Descriptive statistics, reliability coefficient, and correlations among the tasks used in the study.

Tasks	*M*	SD	Reliability	Min–max	2	3	4	5	6
1. Physics skills	26.78	5.61	0.83^a^	15–38	0.28	0.30	0.38	0.22	0.25
2. Verbal working memory span	3.99	0.83	0.89^a^	1–6		0.05	0.12	0.19	0.38
3. Spatial ability (mental rotation)	8.04	3.55	0.93^a^	0–16			0.24	0.12	0.28
4. Raven’s matrices	24.19	5.29	0.74^b^	7–33				0.47	0.33
5. Arithmetic calculation	5.27	2.36	0.76^a^	0–11					0.52
6. Reading comprehension	10.27	3.45	0.96^a^	5–20					

*n = 81, correlation coefficients larger than r = 0.18 are significant at p < 0.05*.

a *Split-half reliability*.

b *Cronbach’s alpha*.

### Multiple Regression Analysis

The regression model, *F*(5, 80) = 4.68, *p* = 0.001, *R*^2^ = 0.238, predicted 24% of the variance in physics. Verbal working memory, spatial processing (mental rotation), and Raven’s progressive matrices emerged as significant predictors. They accounted for 4.5, 4.6, and 5.7% unique variance, respectively, as indicated by their squared partial correlations depicted in the right part of Table 2. The reading and mathematics tasks did not account for any unique variance (*p*’s > 0.05). More detailed information concerning the results of the multiple regression analysis is presented in Table 2.

**Table 2:** Regression analysis of physics skills: the contribution of logical reasoning, verbal working memory, spatial ability, arithmetic calculation, and reading comprehension.

Tasks	*B*	SE	*ß*	*t*	*p*	*pr*^2^^†^
Verbal working memory span	1.556	0.741	0.230	2.102	0.039^*^	0.045
Spatial ability (mental rotation)	0.357	0.169	0.226	2.112	0.038^*^	0.046
Raven’s matrices	0.293	0.124	0.276	2.370	0.020^*^	0.057
Arithmetic calculation	0.054	0.304	0.023	0.176	0.861	0.018
Reading comprehension	0.003	0.213	0.002	0.014	0.989	0.001

† Squared part correlation represents the unique amount of variance accounted for by each predictor. F(5, 80) = 4.68, p = 0.001, R^2^ = 0.238, *p < 0.05.

## Discussion

The present study examined to what extent third grade abilities concerning spatial processing, verbal working memory, and logical reasoning are long-term cognitive predictors of children’s physics skills in sixth grade.

As hypothesized, all three cognitive abilities accounted for variation in sixth graders’ physics skills. Moreover, logical reasoning, spatial processing, and verbal working memory appear to be equally important abilities as they account for similar amounts of unique variance, 5.7, 4.6, and 4.5%, respectively. These amounts of accounted unique variance are rather typical in comparison with previous studies (Mayer et al., 2014; Zhang et al., 2017; Hodgkiss et al., 2018; van der Graaf et al., 2018; Donati et al., 2019). Furthermore, similar to prior research, a large amount of variance remains to be explained as the multiple regression model accounted for only 24% of the variation in physics achievement.

The present findings are important and novel as no prior study has simultaneously examined and observed that logical reasoning, spatial processing, and verbal working memory are unique long-term predictors of children’s skills in physics. As such, they support the notion that physics is a multivariate academic discipline that draws upon numerous cognitive resources (Byrnes and Miller, 2007; Klahr et al., 2011; Ozel et al., 2013; van der Graaf et al., 2016; Vilia et al., 2017; Zhang et al., 2017).

Although, the current overall findings do not correspond entirely with any prior study, parts of the results are interesting in relation to prior research. For instance, the involvement of logical reasoning ability (Raven’s progressive matrices) in 12- to 13-year-old children’s physics performance is consistent with evidence showing that logical reasoning is a key component in order for children and adults to learn about abstract scientific facts, concepts, theories, and applying complex scientific methods (e.g., van der Graaf et al., 2015; Vilia et al., 2017; Berkowitz and Stern, 2018; Donati et al., 2019). On the other hand, it should be noted that Mayer et al. (2014) did not find that 10-year-olds’ scientific reasoning was uniquely supported by logical reasoning.

The finding that spatial processing (mental rotation) emerged as long-term predictor of physics skills in 12- to 13-year-olds is in line with prior evidence of a spatial processing-physics connection in adults (Hegarty and Sims, 1994; Isaak and Just, 1995; Shea et al., 2001; Kozhevnikov et al., 2007; Webb et al., 2007; Wai et al., 2009; Kell et al., 2013).

Moreover, it builds on Mayer et al.’s (2014) and Hodgkiss et al.’s (2018) studies, who found that spatial processing (mental rotation; mental folding) contributes to scientific reasoning in 10-year-olds and physics in 7- to 11-year-olds, respectively. The present findings demonstrate that spatial processing is also a key component in 12- to 13-year-old children’s physics skills. More specifically, the present study and Hodgkiss et al. (2018) and Mayer et al. (2014) show that intrinsic dynamic spatial abilities (e.g., mental rotation, mental folding) underlie 7- to 13-year-old children’s physics skills. Thus, it emphasizes the ability to mentally rotate visual-spatial images when conceptualizing physics phenomena and solving physics problems (Isaak and Just, 1995; Kozhevnikov et al., 2002; Miller and Halpern, 2013). During physics problem-solving, mental rotation processes may theoretically sub-serve the assembly of diverse sources of visual-spatial information into a spatial-schematic image, which has been shown to be critical for success in physics (Kozhevnikov et al., 2002). It should, however, be noted that Zhang et al. (2017) did not obtain any link between spatial processing and physics and chemistry skills in 6-year-old children.

The present result provides further support for the assumption that solving physics problems relies on the flexible and efficient mental workspace referred to as working memory (c.f., Hegarty and Sims, 1994; Isaak and Just, 1995; Kozhevnikov et al., 2007; Hegarty, 2014). Similar to prior studies on children aged 11–16 years and children aged 4–6 years focusing on general science achievement, third grade verbal working memory capacity accounted for individual differences in sixth grade physics achievement (c.f., Jarvis and Gathercole, 2003; Gathercole et al., 2004; St. Clair-Thompson and Gathercole, 2006; van der Graaf et al., 2016, 2018; Donati et al., 2019). The present finding in combination with prior research suggests that verbal working memory is a key component at different developmental stages of science learning, from early preschool stages to middle school stages.

The observed verbal working memory-physics link is theoretically reasonable as physics is a complex academic discipline, involving a complex interplay of processes. Thus, a cognitive system capable of monitoring, coordinating, and executing multiple processes is necessary in order to successfully manage sixth grade physics. For example, physics problems usually entail both linguistic (textual) and visual-spatial information (graphs; diagrams) that has to be related and/or combined in order to solve the problem (Cromley et al., 2013; Bergey et al., 2015). The working memory system should be involved is this process of relating and combining different sources of information.

Contrary to the hypotheses and prior studies (e.g., Maerten-Rivera et al., 2010; Mayer et al., 2014; Barnard-Brak et al., 2017), reading comprehension and arithmetic calculation did not emerge as unique long-term predictors of physics skills in 12- to 13-year-olds. This should not be taken to suggest that reading comprehension is irrelevant to children’s physics learning and development, and that the two STEM disciplines, mathematics and physics, do not share any cognitive processes in general. As a matter of fact, both reading skill and arithmetic skill correlated with physics indicating that they contribute to physics through shared variance. The lack of a unique reading-physics association is probably due to the design of the physics test, which was intended to impose as little linguistic demand as possible. The absence of a unique arithmetic-physics association may suggest that basic calculation is not strongly related to basic physics. However, more advanced mathematics (e.g., geometry, trigonometry, algebra) and physics may very well share underlying cognitive processes.

The multiple regression model accounted for 24% of the variation in physics achievement. Approximately 15% of the 24% was uniquely accounted for by spatial processing, verbal working memory, and logical reasoning; thus, 9% was shared variance. This shared variance indicates that a number of key processes involved in physics are tapped by all five measures. Based on findings from prior research, probable candidates of such key processes might be attention and inhibition control and other executive functions (Rhodes et al., 2014; Zhang et al., 2017; van der Graaf et al., 2018).

### Conclusions, Limitations, and Future Research

The present findings should be interpreted with some care, as the sample size was rather small compared to most prior studies reviewed in the introduction (median sample size = 112). Thus, future studies should replicate the present study with a larger sample. A larger sample should also make it possible to include larger number of cognitive variables. For example, it would be theoretically interesting to include both verbal and visual-spatial working memory tasks, and measures of spatial visualization, spatial perception as well as mental rotation. Then it would be possible to examine the unique relative involvement of different working memory resources and different spatial processing abilities in children’s and adults’ physics skills. Still, this study presents new and theoretically important findings by showing that physics is a multivariate discipline that draws upon numerous cognitive resources. Logical reasoning, verbal working memory, and spatial processing appear to have equally important roles in 12- to 13-year-old children’s physics skills.

### Practical Implications for Teaching

The present study suggests that in order to facilitate children’s learning of physics, the regular science teaching should be supplemented with training of general cognitive abilities. Consistent with prior intervention studies focusing on the STEM complex, mental rotation is a spatial processing ability that should be targeted in such intervention (Uttal et al., 2013; Stieff and Uttal, 2015). Although, the effect of working memory training on science achievement has not yet been studied, the present finding suggests that this training might be a way to enhance children’s physics skills. The fact that working memory training has demonstrated positive effects on mathematics learning, another STEM domain, supports this assumption (Holmes et al., 2009; Loosli et al., 2012; Kuhn and Holling, 2014). Logical reasoning is also an important ability to exercise in order to enhance physics skills. This ability has traditionally been seen as none-malleable, but recent studies suggest that this might not be the case (Buschkuehl and Jaeggi, 2010; Au et al., 2015). Given the present findings and prior interventions studies, the most effective approach to enhance children’s science learning, above and beyond regular science teaching, might be to implement a combination training program that targets all three abilities: logical reasoning, working memory, and spatial processing.

## Data Availability

The datasets generated for this study are available on request to the corresponding author.

## Ethics Statement

This research project (Can we predict 6-year-olds’ future mathematical skills? A longitudinal study on mathematical difficulties) has been approved by the regional ethics committee in Linköping, Sweden (Protocol number 33-09). The children were recruited by means of a letter of consent that they brought home to their parents from school. All children with written parental consent were included.

## Author Contributions

UT contributed by designing the study, collecting data, performing data analyses, and writing a draft of the manuscript. LO contributed by collecting data and writing a draft of the manuscript. KS and MS contributed by designing the study and writing a draft of the manuscript. RÖ contributed by collecting data, performing data analyses, and writing a draft of the manuscript.

### Conflict of Interest Statement

The authors declare that the research was conducted in the absence of any commercial or financial relationships that could be construed as a potential conflict of interest.
